# Technology to the rescue: how to uncover the role of transposable elements in preimplantation development

**DOI:** 10.1042/BST20231262

**Published:** 2024-05-16

**Authors:** Lauryn A. Deaville, Rebecca V. Berrens

**Affiliations:** 1 Institute for Developmental and Regenerative Medicine, Oxford University, IMS-Tetsuya Nakamura Building, Old Road Campus, Roosevelt Dr, Oxford OX3 7TY, U.K.; 2 Department of Paediatrics, Oxford University, Level 2, Children's Hospital, John Radcliffe Headington, Oxford OX3 9DU, U.K.; 3 MRC Weatherall Institute of Molecular Medicine, Oxford University, John Radcliffe Hospital, Oxford OX3 9DS, U.K.

**Keywords:** epigenetics, gene regulation, preimplantation development, transposable elements

## Abstract

Transposable elements (TEs) are highly expressed in preimplantation development. Preimplantation development is the phase when the cells of the early embryo undergo the first cell fate choice and change from being totipotent to pluripotent. A range of studies have advanced our understanding of TEs in preimplantation, as well as their epigenetic regulation and functional roles. However, many questions remain about the implications of TE expression during early development. Challenges originate first due to the abundance of TEs in the genome, and second because of the limited cell numbers in preimplantation. Here we review the most recent technological advancements promising to shed light onto the role of TEs in preimplantation development. We explore novel avenues to identify genomic TE insertions and improve our understanding of the regulatory mechanisms and roles of TEs and their RNA and protein products during early development.

## Introduction

**Figure 1.**
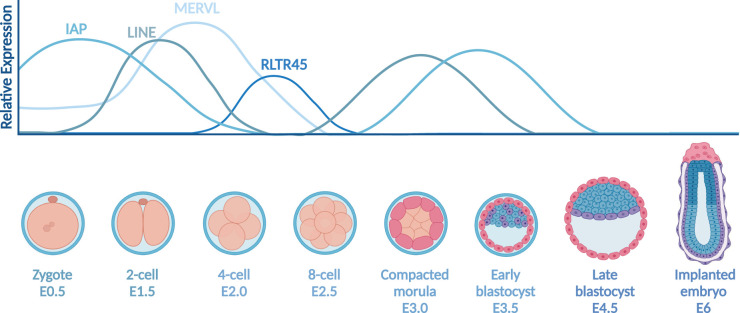
Schematic of TEs expressed at stages of preimplantation development. Adapted from [5,6]. Created with BioRender.com

During preimplantation development, the zygote undergoes meticulously regulated cell divisions accompanied by global epigenetic reprogramming and activation of the zygotic genome (reviewed in [1]). Simultaneously, significant changes in gene expression facilitate lineage specification, forming both the inner cell mass and the trophoblast tissue (reviewed in [2]). Lineage specification is essential for implantation and viability of the developing organism. Cells produced during preimplantation contribute to the development of all differentiated cell types in an organism. The expression of transposable elements (TE) plays an important role in preimplantation development, and the intricate relationship between TE expression and this phase of early development has been extensively reviewed in [3–5] (Figure 1). However, repetitive genetics and low cell-numbers present unique problems to researchers studying TEs in preimplantation.

TEs are mobile genetic elements, a quirk which has resulted in hundreds of thousands of copies of TEs with near-identical sequences. Over time, these sequences acquire mutations, diversifying loci. Therefore, with sufficient sequencing depth, many older families of TEs can be studied using canonical short-read sequencing technologies [7]. However, the lack of sequence diversity in younger TEs makes only ∼10% of the youngest loci mappable [7,8] This bias in TE research often underestimates the activity and roles of young, retrotransposition active TEs [8].

**Figure 2.**
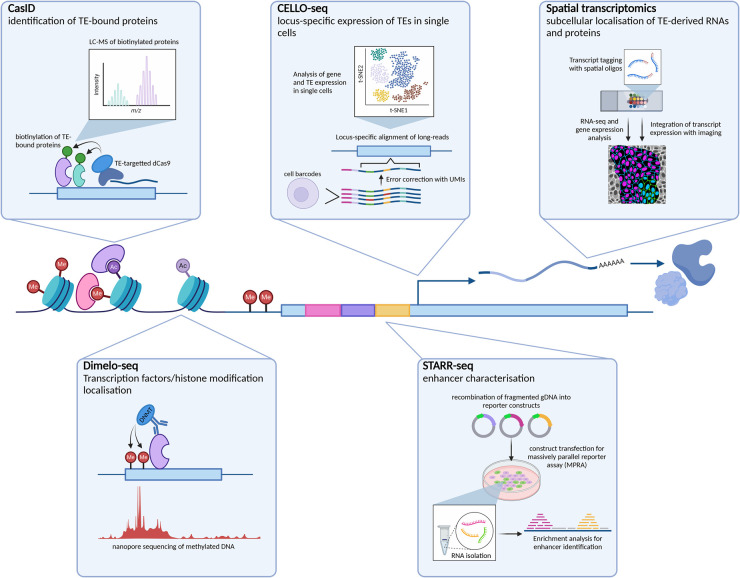
Technology for Transposable elements — Schematic of key technologies for the study various aspects of TE and preimplantation biology. CasID: Cas9-targeted biotinylation of TE-bound proteins, followed by streptavidin pulldown and Liquid Chromatography-Mass Spectrometry (LC-MS) for protein identification [11]. CELLOseq: addition of cellular barcodes and unique molecular identifiers (UMIs) enables single-cell long-read RNA sequencing for unique mapping of TE-derived transcripts [8]. Spatial Transcriptomics: hybridisation of unique oligos across the sample prior to imaging and RNA isolation enables coupling of imaging with transcriptomic analysis [12]. DiMeLo-seq: Antibody-targeted DNA methyltransferases result in enrichment of m6dA over protein-bound regions which can be resolved with ONT direct DNA sequencing [13]. STARR-seq: genome fragmentation and generation of many reporter constructs is followed by RNA extraction and enrichment analysis for enhancer identification [14]. Created with BioRender.com.

Obtaining embryo samples from both human and mouse is difficult, with each approach imposing different challenges on researchers. Human studies are halted both by ethical constraints, and embryo availability and quality. For mouse work, technical challenges impose the main roadblock. Specifically, throughput is limited to tens of embryos per mouse, and isolation, culture, and experimentation steps result in losses to this already minimal material. Due to these limitations many existing techniques are only available to use in mouse or human pluripotent embryonic stem cells (ESCs), derived from the inner cell mass of the blastocyst. ESCs can be cultured in conditions mimicking various stages of embryo development, and advancements to 3D stem-cell culture and embryo approaches are beginning to provide more diverse embryo models for both human and mouse [9,10]. Using these systems enables the application of existing high-throughput techniques to the study of preimplantation, which have previously been inaccessible due to cell-number constraints. Here, we review existing and novel methodologies with potential to answer open questions surrounding TEs in preimplantation development (Figure 2).

## Exploring embryo alternatives

While many existing technologies to study genetics are now able to be applied to the study of TEs, the uniquely low cell numbers in preimplantation development mean many questions remain unanswered. However, novel *in vitro* embryo and organoid culture systems will enable exploration of developmental processes and genetic mechanisms without the limitations of traditional mouse models. By eliminating the uterine barrier, *ex vivo* systems enable mechanistic interrogation of pre- and post-implantation embryo development in mammals [15–17]. For example, *ex utero* manipulation enables injection of CRISPR proteins directly into zygotes to observe effects of genetic knockouts (KOs) or knockdown (KD) [18]. Such culture systems have already been harnessed to tackle questions in symmetry breaking and germ-layer specification in mice [15,16]. Similarly, human embryonic-like structures called blastoids can be derived from human ESCs, enabling dissection of early human development in the absence of maternal tissues[19,20]. Further optimization and characterization of these systems is required to fully validate the use of gastruloids and blastoids as a model system for preimplantation embryos. However, with the help of *in vivo* validation, these *ex vivo* systems provide a valuable opportunity to conduct higher input experiments and functional screens of TEs in preimplantation; which we will explore in this review.

## What can we learn from looking closer at TE DNA sequences?

Unlike in somatic cells, novel TE insertions arising during preimplantation have the potential to propagate through the germline and contribute to heritable genetic variation in subsequent generations. Their intrinsic drive for self-preservation is thought to promote TE expression prior to primordial germ cell (PGC) specification [21]. Furthermore, TE activity in oogenesis and spermatogenesis can result in insertions caried through to the zygote [22,23]. High-fidelity genome assemblies are crucial for identifying such transposons insertions. This is especially important with regards to younger TEs. Young TEs exhibit high sequence similarity as they remain retrotransposition active, creating novel insertions in different populations, mouse strains, and cell lineages [24–26]. A recent comparison identified strain-specific variations in retrotransposon abundance and activity in mice [27]. For example, long interspersed nuclear element 1 (LINE1) are more abundant in the CB57BL/6 compared with the 129 strain of mice. Despite these variations, many publications continue to use CB57BL/6-derived genome assemblies to study TEs in 129-derived mouse ESCs [28]. This may result in wrongly assigned TEs causing inappropriate assumptions about genomic context, and ultimately roles of TEs. Recent advancements in long-read sequencing technologies, such as PacBio and Oxford Nanopore Technology (ONT) sequencing, have significantly enhanced genome assemblies, particularly in repetitive regions, for example the X chromosome [29,30]. Further advantages for identifying TE insertions might arise through ONTs adaptive sampling. Adaptive sampling allows real-time rejection of undesired sequences by reversing polarity in the nanopore [31,32]. Selection of molecules based on genomic co-ordinates may allow higher fidelity reconstructions of full-length TEs. Although whole genome sequencing technologies can require input of ∼100 000 cells, researchers should consider using *in vitro* models to generate their own genome assemblies to study TEs [27,33]. For example, recent telomere to telomere assemblies may give important insights into sex specific roles of TE insertions in early development [29].

Following identification of TE insertions, exploration of the DNA sequence allows elements to be classified based on their mechanism of transposition, evolutionary age, and sequence similarity (reviewed in [34]). LINE1s for example, can be divided based on the presence of different monomer sequences within the promoter [35,36]. A recent study found that changes in monomer structure of LINE1s can influence nearby reporter gene expression [37]. While we know that LINE1s are expressed in preimplantation development, analysis of monomer structures in expressed LINE1 promoters may decode the mechanism of locus-specific expression. However, expression does not guarantee retrotransposition. Characterizing retrotransposition activity of younger insertions necessitates retrotransposition assays, currently confined to *in vitro* conditions using mouse ESCs [31,32,38]. The exploration of alternative culturing systems or embryos may hold potential for expanding the scope of these assays [39–41].

Many TE families are also predicted to possess enhancer activity; promoting transcription of their own RNA as well as other genes [42–44]. The multifaceted roles of TE-derived enhancers have been reviewed extensively in [45]. However, an open question is whether specific sequences within TEs predispose TE loci to function as cell-type specific enhancers compared with other loci. Existing methods like STARR-seq can be used to test whether a minimal core sequence of a TE loci is sufficient to act as an enhancer [14]. STARR-seq enables a broad-scale examination of enhancers in the genome, identifying regulatory motifs through reporter gene expression. STARR-seq has been previously used to identify bona fide TE enhancers in cancers [46] and human ESCs [47] and, as *in vitro* models improve may facilitate identification of minimal sequence components of TE loci that control developmental gene regulatory networks throughout the course of development, where use of this technique has been limited by low cell numbers.

## How do TEs interact with their chromatin environment?

TE enhancers mediate genomic long-range regulatory interactions. Studies illuminated how expression of enhancer RNA and upstream antisense RNA from complementary Alu sequences can modulate enhancer-promoter interactions of oncogenes, underscoring the profound regulatory potential of TEs in chromatin dynamics [48]. Our understanding of TE-mediated chromatin interactions in embryos remains limited, and the influence of TEs on chromatin architecture during preimplantation is still discussed. For example, LINE1 RNA itself seems to be able to influence epigenetic remodeling through recruitment of NUCLEOLIN and KAP1 [49]. Adaptation of high-resolution capture techniques like Micro Capture-C could allow investigation of interactions of individual loci [50]. For example, using probes against regions surrounding loci of interest was employed to profile TE interactions in HeLa cells, identifying TE interactions using proximal unique sequences [51]. Using a similar strategy may enable exploration of individual TE-derived *cis*-regulatory elements and their genic targets during preimplantation. Additionally, techniques such as Pore-C, which link several genomic loci together prior to long read sequencing, may enable dissection of complex interactions of TE loci with multiple genomic locations [52]. This could shed light on the role of TEs in the compaction of chromatin in the early embryo [53].

Specific motifs within TEs enable binding of cell-type specific transcription factors, contributing to their activities as regulatory elements for other genes. For example, binding of OCT4, KLF4 and KLF17 to a long terminal repeat (LTR) of a human endogenous retrovirus (ERV) K is thought to contribute to hominoid-specific patterns of embryonic gene expression [54–56]. More recently, 5′ UTRs of young LINE1s bound by elongation factor ELL3 were shown to act as enhancers for *Act3*. ACT3 is necessary for activation of a key kinase pathway, providing an interesting link between TE expression and developmental signaling [57]. As well as *trans*-acting factor binding, TE enhancer activity can also be determined by the presence of specific epigenetic modifications. For example, H4K16ac activates transcription of TEs to enable enhancer activity in human ESCs [47]. Studying the role of TEs as enhancers in early development will enable us to understand whether TEs are involved in whole gene regulatory networks or mostly control individual nearby genes.

Next to contributing to their own regulatory functions, epigenetic modifications and binding of trans-acting factors also regulates expression of TEs. For example, deposition of the repressive histone mark H3K9me3 on LTRs leads to stage-specific transcriptional silencing of TEs in eight-cell and blastocyst stage human preimplantation embryos [58]. Further examples have shown increased chromatin accessibility and allele-specific histone modifications suggesting transcriptional activity over TE families during preimplantation development [59–61]. Using updated mapping approaches, analysis of locus-specific transcription of TEs may be possible using these datasets, however insights into young TE families may be limited by short-reads and low coverage. Alongside epigenetic modifications, association of proteins with TE DNA is also known to contribute to their regulation. For example, LINE1s commonly associate with the transcription factor YY1. Interestingly, while in humans, YY1 binding has been shown to silence LINE1 expression; in mice, YY1 binding stimulates expression from LINE1 [60,61]. Alongside YY1, many TEs are known to experience cell-type specific regulation of gene expression by other zinc-finger nucleases (ZNFs) [55,62,63].

Current state-of-the-art techniques for assessing the enrichment of epigenetic modifications and DNA-bound proteins, such as ChIP-seq, are powerful but encounter limitations in *in vivo* work due to the substantial cell numbers required for high-quality data. This limitation poses a challenge, particularly in the context of preimplantation studies where samples are limited. While low-input alternatives such as CUT&RUN or CUT&TAG are improving in quality, the use of short-read sequencing constrains their applicability to exploring protein interactions with TE families [64,65]. Several advanced analysis approaches have enabled robust analysis of TEs using short read data (reviewed extensively in [7]). Building on this, deep learning holds promise for unique mapping of reads from existing short-read experiments. For example, MATES uses adjacent read alignments surrounding the TE locus to allocate multi-mapping reads to specific loci of TEs in single-cell data [66]. MATES may be particularly useful for re-analysis of existing single-cell preimplantation data from [67–70], reducing the costs associated with data generation. Using such algorithms may extract novel information about locus-specific histone modifications over TEs in single-cells. However, analysis approaches are still limited by sequencing depth and, in very old datasets, by read length and quality, encouraging the use of novel long-read sequencing approaches.

Novel technical approaches, such as ONT long-read sequencing, offer a promising solution to limitations in existing genomics methods. Direct sequencing of native DNA allows identification of base modifications, enabling detection of DNA modifications such as 5mC and m6dA. Co-option of DNA modifying enzymes allows investigation of DNA-protein interactions as well as chromatin features over repetitive regions. DiMeLo-seq uses antibodies to direct DNA adenine methyltransferases to proteins of interest. DiMeLo-seq has provided valuable insights into protein dynamics within mammalian centromeres holding promises for TE research [13]. Similarly, DamID uses DNA adenine methyltransferases, however these are targeted via fusion proteins [71,72]. Another example is STAM-seq, which identifies DNA methylation and open chromatin within repetitive regions [73]. While the requirement for a million cells as input limits the use of these techniques *in vivo*, techniques such as DiMeLo-seq and STAM-seq could be adapted to provide insight into locus-specific changes in chromatin modifications and accessibility during pluripotency exit in *in vitro* cultures such as gastruloids, for example to study the zygote to blastocyst transition. With DiMeLo-seq, it is possible to explore protein binding density on a single chromatin fiber from a single cell, enabling exploration of heterogeneity in transcription factor distribution across individual TEs in single cells in the developing embryo.

Many factors associated with TE function in preimplantation remain elusive. An emergence in dead Cas9-based targeting technologies allows researchers to identify novel proteins binding to TE DNA. Via endonuclease-inactive Cas9, gRNAs target modifier proteins to specific TE families to identify novel protein candidates involved in TE regulation or function. Such technologies have already improved understanding of TE DNA-protein interactions. In human ESCs, CARGO-BioID identified the RNA *N*^6^-methyladenosine (m6A) reader, YTHDC2, to occupy genomic loci of LTR7/HERV-H through its interaction with m6A-modified HERV-H RNAs. YTHDC2 further recruited the DNA demethylase TET1 thereby preventing epigenetic silencing of TE loci which inhibited neural differentiation of human ESCs [74]. A similar technology, CasID, identified TNRC18 as a chromatin-associated regulator of ERVs. Specifically, TNRC18 binds H3K9me3 and recruits co-repressors like HDAC–SIN3–NCoR complexes to silence ERV1 expression [11]. So far, these techniques have been successful in studying TEs at the family-level. Improvements to genome assemblies and gRNA specificity targeting upstream of the promoter of an individual TE locus may enable a locus-specific TE view of protein binding in the future.

Covalent epigenetic modifications to DNA such as 5mC act as a critical repressor of TE expression [36,75–77]. Direct sequencing of native DNA also provides a unique opportunity to explore changes in DNA methylation of TE loci. The ability to also measure 5mC as well as 5hmC simultaneously eliminates the need for the conventionally used bisulfite conversion [78,79]. Long-read sequencing identified variation in *de novo* methylation of LINE1 loci, which may allow transcription of individual loci during mouse ESC differentiation [36]. Furthermore, ONT sequencing identified *de novo* activity of DNA methyltransferase DNMT1 targeted to IAP retrotransposons, which silences IAP expression in mouse ESCs [80]. ONT sequencing also allows to explore other base modifications, such as the selective oxidation of 5mC to 5hmC. Oxidation of 5mC to 5hmC occurs in the paternal genome during the earliest stages of preimplantation [81,82]. Several studies have explored TET3 activity on LINE1s, however, locus-specific data on 5hmC distribution during preimplantation from direct DNA sequencing could deepen our understanding of the role of 5hmC in TE regulation during early development [83,84]. This may reveal how methyltransferases are targeted to induce changes in DNA methylation at the TE locus level [84].

## What else can we learn from TE expression?

While exploring epigenetic changes around TEs is insightful, often the link between chromatin modifications and TE expression is unclear. Many studies focus on the loss of repressive marks during preimplantation, which alone is insufficient to answer outstanding questions of TE dynamics [76,85–88]. For example, a recent study on locus-specific DNA methylation shows not all demethylated loci are expressed in human ESCs [89]. However, after extensive research, it is known that TEs are expressed during specific stages of preimplantation, prior to silencing in most somatic cells [90] (Figure 1). For example, in the mouse genome, elevated transcription of MERVL and MaLR elements is a known marker of zygotic genome activation (ZGA) [91–94]. Expression of ERVs has also been identified in human preimplantation embryos, starting from ZGA at the eight-cell stage [56,90]. Alongside ERVs, the earliest expressed retrotransposons are LINE1s, which are detected first in the zygote. While expression peaks in the two-cell stage, LINE1s remain transcriptionally active throughout embryonic development [95].

Although family-level expression dynamics of TEs are well characterized, the limitations of short-read transcriptomic data and imaging approaches mean it remains a mystery if loci are expressed preferentially, especially younger elements with more similar sequences. Expression of young TEs is often underestimated using only unique mapping reads [8,96]. Computational methods try to overcome this by assigning reads to specific loci depending on how many reads each locus from a subfamily overlaps [97,98]. Several studies have used short-read single-cell data to study TE expression in single cells, either to assess family level expression of TEs [99] or study TE expression in groups of cells in a range of systems [99–101]. For example, combination of mapping approaches with genomic classification has enabled locus-resolved TE expression in bulk RNA-seq data in zebrafish [100]. Single-cell RNA-seq in mouse and human embryos may also reveal allele-specific variation in locus-specific TE expression [100]. Advanced analysis approaches of short-read data ensure accuracy of TE expression, and begin to provide locus-specific and single-cell resolution of TE dynamics. However, the youngest TEs lacking uniquely mappable sequences from short-read data continue to limit the applicability of such approaches.

Alternatively, current advances in long-read RNA-seq methodologies hold great promise for locus-specific expression analysis. PacBio sequencing combined with target enrichment to investigate LINE1 expression, focusing on 5′ UTR primers to discern transcription initiation from TE promoters [102]. However, these methods lack single-cell resolution. CELLO-seq, offers a unique advantage by providing long-reads with error-correction, facilitating precise mapping of TE transcripts at the locus level within single cells [8]. Single-cell long-read RNA-seq proves particularly beneficial for examining preimplantation stages, allowing for the exploration of heterogeneity in TE expression within individual cells of the embryo. Potential improvements, such as multiplexing of samples and automation of the pipeline hold promise for enhancing throughput in TE analysis.

LTRs of ERVLs also give rise to TE-derived isoforms that are expressed throughout ZGA and long-read single-cell RNA-seq will enable identification of TE-derived isoforms throughout early development [103]. Exploration of chimeric TE-gene transcripts raises questions about their authenticity, potentially being either artifacts of PCR or true structural variations [104,105]. Overlap with known short-read data, utilization of splice sites, and correlation with chromatin data could enhance the identification of genuine TE transcripts [106]. Additionally, investigating native transcription with techniques like TT-seq involving 4-Thiouridine incorporation may distinguish actively transcribed loci from residual nuclear RNA [107]. However, the inability of reverse transcriptases to work with highly structured RNA and biases introduced during PCR amplification highlight the need for alternative methods without need for amplification.

Direct RNA-seq emerges as a reliable method for qualitatively measuring locus-specific TE expression without the need for PCR amplification. While direct RNA sequencing requires high input and high-quality RNA, which is often not feasible from single embryos, this method may be applicable to *in vitro* samples. Direct RNA-seq also enables exploration of the role of RNA modifications in TE RNA activity. Notably, m6A was shown to mark retrotransposon-derived RNAs in oocytes and preimplantation embryos, including MTA and MERVL during ZGA at the two-cell stage [108]. This modification extends to young LINE1s in later stages, suggesting a role in RNA stability, localization, and translation of these transcripts during the transition from totipotency to pluripotency [108]. However, the protocol remains limited by 3′ biases and difficulties in detecting RNA modifications remain. Limitations aside, new flow-cell chemistry together with more data for improved training of base callers may identify a role for RNA modifications in TE-RNA stability and improve understanding of the function of TE RNAs in preimplantation development. To date, the sequence or RNA modifications of currently active LINE1s in the human genome remain unknown. Further investigation is needed into the impact of TE-RNA modifications on early embryo development.

## What is the functional role of TE expression?

Various approaches have been used to understand the regulation of TEs, but most are correlative. As with genes, approaches such as KD or KO systems are essential to study function. Large-scale CRISPR screens involving Cas9-mediated KO of epigenetic regulators were able to identify novel protein factors involved in retroelement silencing alongside SETDB1 in mouse ESC [109]. Similar screens identified factors involved in retrotransposition [110,111]. CRISPR screens could prove useful for the study of TE loci themselves, rapidly providing insight into the contributions of many different TEs into gene regulation in mouse ESC. On a smaller scale, KO of an individual LTR10A-ENG in human trophoblast stem cells identified its role as an enhancer for the growth factor receptor ENG. Inappropriate activation of this enhancer is thought to contribute to preeclampsia, another example highlighting the importance of TEs in preimplantation [112]. However, KO approaches can cause disruption by removing large parts of the genome. This is a particular issue when studying younger, intact elements that can be over 6 kb in length.

Alternative methods aim to epigenetically silence genomic locations or remove RNAs to recapitulate TE silencing. Epigenetic silencing targeted using transcription activator–like effector (TALE) proteins or by antisense oligos showed precise expression of LINE1 at the two-cell stage is involved in progression to the blastocyst stage [49,113]. However, more recently co-option of the CRISPR system (termed CRISPRi) has improved targeting of sequence-specific silencing. CRISPR inactivation of MERVL highlighted that their transcription is essential for preimplantation [114]. While these approaches might be favorable, concerns arise with KD due to spreading of chromatin modifications causing alterations to surrounding genetics [115]. It will be necessary to constrain KD effects and targeting to ensure KD individual loci instead of whole TE families, because individual loci of TE families might overlap with genic regions, and it remains unclear how much of the KD can be attributed specifically to the TE expression.

## What are the roles of TE RNA and protein?

Both RNA and protein products of TE expression are thought to contribute to preimplantation development, and several methodologies can be applied to explore their roles. Imaging techniques have been integral to ZGA studies, with single molecule imaging using the MS2 loop system combined with HALO-tag technologies emerge as novel methods to infer transcription dynamics in early embryos [116], reviewed in [117]. Applying this methodology, researchers found cell cycle dependent subcellular organization of LINE1 RNA in HEK293T cells [118]. Application of imaging technology may provide answers to other outstanding questions surrounding TE RNA biology. Both coding and non-coding nascent RNAs, including those produced by retrotransposons, may play an important role in forming transcriptional condensates in development and disease contexts [119]. Such condensation of LINE1 is also identified as a critical factor for retrotransposition [120]. Studying condensates using single molecule imaging technologies in early embryos could give important insights into the timing of retrotransposition events, as well as the role of TE-derived transcriptional condensates in preimplantation [119].

RNA fluorescence *in situ* hybridization (RNA-FISH) has served as a foundational technique in early embryo investigations. The expression of TEs such as IAPs and LINE1s during ZGA, were initially characterized through RNA-FISH methods [95]. Multiplexing of RNA-FISH probes has enabled the development of spatial transcriptomics, employing techniques like MERFISH, 10× Genomics, and NanoString [12,121,122]. Combining TE-specific probes with epigenetic markers and key ZGA transcripts holds great potential for understanding single-cell regulation within developing embryos. However, application of spatial transcriptomics to TE RNAs remains a challenge due to the requirement for robust and specific probes and multiplex readouts.

Spatial approaches are not limited to RNA, with spatial proteomics emerging as a promising avenue for the study of protein localization in preimplantation [123]. Spatial proteomics could reveal heterogeneity of TE protein translation between the inner and outer cells of eight-cell stage embryos, or differences in subcellular protein localization. However, recent work on LINE1s in cancer underscores the need for careful characterization of TE antibodies to ensure specificity [124]. Such protein tags have also been used in conjunction with retrotransposition assays, potentially facilitating a more precise understanding of protein localization during retrotransposition [38]. Advancements to spatial transcriptomics, proteomics, and advanced imaging may provide a unique perspective on the interaction of TE RNA and protein with other cellular pathways during preimplantation. For example, resolution of ZFPs alongside TE transcripts during preimplantation development could give important insights into the temporal regulation of TE expression.

**Table 1.** Techniques and their description with limitations and open questions in preimplantation they could answer

Technique	Description	Limitations	Questions to be answered
Direct DNA-seq (ONT)	Long read direct DNA sequencing by Nanopore by sequencing directly isolated DNA. This allows to identify TE insertions as well as characterize their 5mC and 5hmC levels.	×10^5^ cells *in vitro*	When do de novo TE insertions happen in the genome? Are these loci differentially methylated?
STARR-seq	Self-transcribing active regulatory region sequencing, a method for identifying and mapping active enhancer elements by capturing nascent RNA transcripts synthesized from active regulatory regions.	×10^6^ cells *in vitro*	Can we identify minimal core sequences in TE subfamilies that act as enhancers at different stages of preimplantation development?
Micro Capture-C	Cross-linking agents fix chromatin structure ahead of proximity ligation. Probe-mediated pull-down is used to capture chromatin interactions for regions of interest. Captured chromatin fragments are sequenced to identify interacting genomic regions.	×10^6^ cells *in vitro*	Do TE loci exhibit enhancer function important for the first cell fate choice early in two cell stage embryo changing gene expression patterns at eight cell stage?
Pore-C	A chromatin conformation capture technique that ligates multiple contact sites prior to nanopore sequencing to analyze chromatin interactions, enabling the study of complex chromatin folding and organization at high resolution.	×10^6^ cells *in vitro*	Do TE loci of the same family interact with each other to build enhancer hubs? Are they involved in droplet formation and play a functional role in pluripotency?
MATES	Computational method that uses adjacent read alignments surrounding the TE locus to allocate multi-mapping reads to specific loci of TEs in single-cell data.	*In silico*	Is there heterogeneity of histone modifications over individual loci of TEs in single cells of the early embryo?
CUT&RUN/CUT&TAG	CUT&RUN involves targeted cleavage of chromatin-bound proteins using an antibody and micrococcal nuclease (MNase), before sequencing of DNA fragments. CUT&Tag uses an antibody-targeted Tn5 transposase to directly tag the protein-bound DNA before sequencing.	×10^4^ cells *in vitro*/*in vivo*	Do specific transcription factors regulate locus-specific TE expression in early embryos?
DiMeLo-seq	Profiling DNA-protein interactions at single-cell resolution by using antibodies to specifically target DNA adenine methylation to protein-bound sites. Nanopore sequencing identifies DNA methylation patterns in individual cells.	×10^6^ cells *in vitro*	Do individual TEs show accumulation of specific histone modifications during pluripotency exit?
STAM-seq	Tn5-based ATAC-seq with molecular identifiers, a method for profiling chromatin accessibility at single-cell resolution, allowing the study of regulatory elements in individual cells.	*In vitro*/*in vivo*	Is accessibility of individual TE loci heterogenous in preimplantation embryos?
CARGO-BioID	A bait protein fused to a promiscuous biotin ligase (BioID) is expressed in cells, and biotinylates neighboring proteins. Biotinylated proteins are captured by streptavidin beads and identified using mass spectrometry.	×10^6^ cells *in vitro*	Can we identify new interactors of LINE1 RT to understand whether any host proteins are involved in LINE1 retrotransposition in the early embryo?
CasID	CRISPR-Cas9-targeted labeling of proteins bound to specific DNA sites, enabling the identification of protein-DNA interactions.	×10^6^ cells *in vitro*	Do different TE loci show accumulation of distinct protein interactors? Does this impact TE expression?
CELLO-seq	Single-cell long-read RNA sequencing, using cellular barcodes and UMIs to enable single cell and single loci resolution of TE expression.	*In vivo*	Which TE loci are expressed in single cell blastomeres? Can we detect novel TE-derived isoforms or allele specific TE expression?
TT-seq	Transcriptional run-on followed by high-throughput sequencing, a technique for measuring nascent transcription rates by labeling and sequencing newly synthesized RNA molecules with thiouridine from actively transcribing genes.	×10^7^ cells *in vitro*	Can we identify actively transcribed TE loci? Does the stability of different TE RNAs vary?
Direct RNA-seq	Direct sequencing of RNA without amplification by Nanopore. This enables transcriptome analysis as well as identification of RNA-modifications, such as m6A.	×10^6^ cells *in vitro*	Can we detect novel TE-derived isoforms? What is the distribution and impact of RNA modifications on TE-derived transcripts?

## Perspectives

- The exploration of TE DNA, chromatin, expression, and products in the context of preimplantation development is a multifaceted endeavor. The highly repetitive and mobile qualities make TEs perhaps the most unique genetic elements. Unique genetics alongside the reduced cell numbers available have limited research into TEs in preimplantation. A broad range of recent technological advancements provide the opportunity to explore the roles of TEs.
- TEs are frequently studied as groups of repetitive elements. KD of several TE families in preimplantation embryos have revealed their importance in this phase of development. However, these KDs can affect up to 10% of the genome and, like many other technologies, are limited to investigations of TE families. Since, a technological revolution has enabled a new focus of TE research, at the level of individual loci. Increasingly, such research highlights heterogeneity in the activity, environment, and functions of different TE loci, underlining the importance of a locus-specific view.
- We encourage researchers to move away from the traditional view of TEs as groups of repetitive elements, and to focus on their uniqueness. Discrepancies in TE prevalence among mouse strains emphasizes the need for caution when using genome assemblies. Implementation of long-read technologies will afford a locus-specific view of TE regulation and function and allow a more accurate view of novel TE insertions and their environment. Furthermore, single-cell approaches will teach us about heterogeneity of TEs between cells of the early embryo. In the future, combining these technologies with advancements in *in vitro* methods to study mouse as well as human development will provide unprecedented insights into the role of TEs in preimplantation development.

## Open Access

Open access for this article was enabled by the participation of University of Oxford in an all-inclusive *Read & Publish* agreement with Portland Press and the Biochemical Society under a transformative agreement with JISC.

## Abbreviations

ERV: endogenous retrovirus
ESC: embryonic stem cell
KD: knockdown
KO: knockout
LINE1: long interspersed nuclear element 1
LTR: long terminal repeat
ONT: Oxford Nanopore Technology
PGC: primordial germ cell
RNA-FISH: RNA fluorescence *in situ* hybridization
TALE: transcription activator–like effector
TE: transposable element
UMI: unique molecular identifier
ZGA: zygotic genome activation
ZNF: zinc-finger nucleases
